# Supplementary material for: A Fast and Selective Approach for Profiling Vicinal Diols Using Liquid Chromatography-Post Column Derivatization-Double Precursor Ion Scanning Mass Spectrometry
Source: Molecules. 2022 Jan 3;27(1):283. doi: 10.3390/molecules27010283 (PMC8747065; doi:10.3390/molecules27010283)
Supplement: Supplementary file 1 [file molecules-27-00283-s001.zip › molecules-1468227-revised-2-sup.pdf]

Supplemental Information:

A fast and selective approach for profiling vicinal diols using liquid chromatography-post column derivatization-double precursor ion scanning mass spectrometry

Debin Wan,[1] Christophe Morisseau[1], Bruce D Hammock,[1] and Jun Yang[1,*]

[1] Department of Entomology and Nematology, and UCD Comprehensive Cancer Center, One Shields Avenue, University of California, Davis, Davis, CA 95616, USA

* Correspondence: junyang@ucdavis.edu; Tel.: +1 530-752-5109

Table S1. HPLC gradient to separate 1,2-cis-diols

Total Time (min)	Flow Rate (μL/min)	B (%)
0	250	35
0.25	250	35
1	250	45
3	250	55
8.5	250	66
12.5	250	72
15	250	82
16.5	250	95
18	250	95
18.1	250	35
21.5	250	35

Table S2. Optimized mass spectrometric parameters on Q-trap

Parameters	Value
CUR	25 psi
TEM	600 °C
GS1	50 psi
GS2	60 psi
iHe	ON
CAD	Medium
IS	5500
EP	10

Table S3 Optimization of modifiers for post column derivatization

Modifier	Signal intensity (cps)
0.5% Acetic acid, pH 6	265
5% NH_3-H_2O, pH 9	930
water	1310

Table S4 Reproducibility of the developed method

Concentration level (ng/mL)	Intra-day Precision	Inter-day Precision
50	13.80%	12.60%
500	3.80%	4.50%
5000	2.50%	7.90%

Note: the data are calculated using the response of 14,15 DiHETrE-BPBA ester to d11-11,12 DiHETrE-BPBA ester.

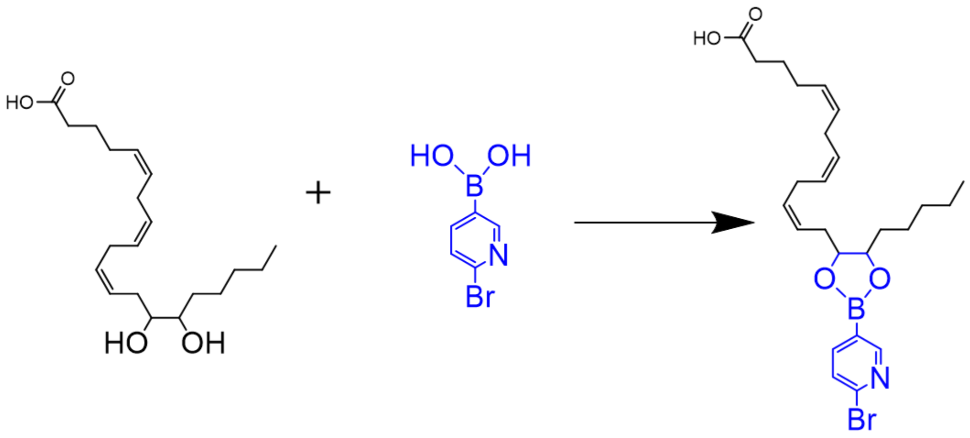

Figure S1: An example (14,15 DiHETrE) of the derivatization of vicinal diolS by BPBA.

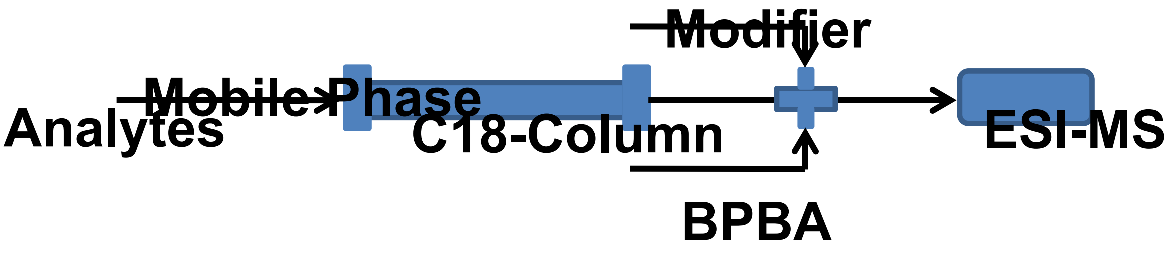

Figure S2: Scheme of the post column derivatization configuration used.

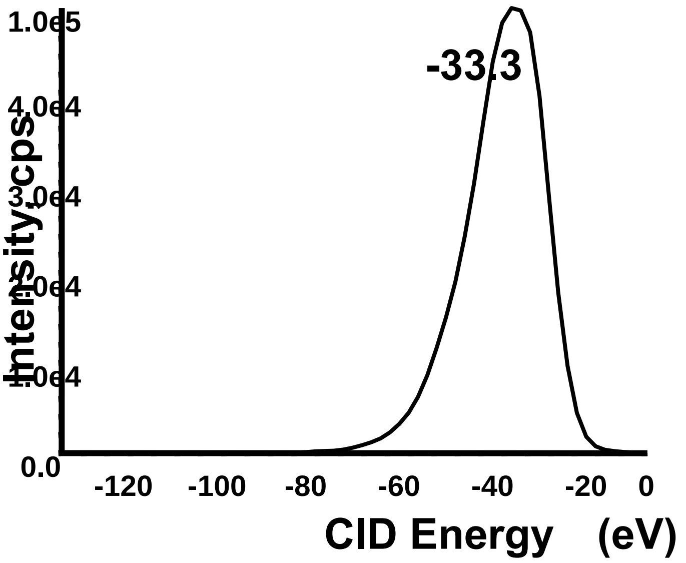

Figure S3: Optimized CID energy for each compound.
